# Protocol for Genome Editing to Produce Multiple Mutants in Wheat

**DOI:** 10.1016/j.xpro.2020.100053

**Published:** 2020-06-13

**Authors:** Fumitaka Abe, Yuji Ishida, Hiroshi Hisano, Masaki Endo, Toshihiko Komari, Seiichi Toki, Kazuhiro Sato

**Affiliations:** 1 Division of Basic Research, Institute of Crop Science, NARO, Tsukuba 305-8518, Japan; 2 Plant Innovation Center, Japan Tobacco Inc., Iwata 438-0802, Japan; 3 Institute of Plant Science and Resources, Okayama University, Kurashiki 710-0046, Japan; 4 Division of Applied Genetics, Institute of Agrobiological Sciences, NARO, Tsukuba 305-8634, Japan

## Abstract

Here, we describe a protocol for producing multiple recessive mutants via genome editing in hexaploid wheat (*Triticum aestivum*) cv. Fielder. Using *Agrobacterium*-delivered CRISPR/Cas9 and three sub-genome-specific primer sets, all possible combinations of single, double, and triple transgene-free mutants can be generated. The technique for acceleration of generation advancement with embryo culture reduces time for mutant production. The mutants produced by this protocol can be used for the analysis of gene function and crop improvement.

For complete details on the use and execution of this protocol, please refer to Abe et al. (2019).

## Highlights

• A method for producing genome-edited multiple-recessive mutants in wheat
• Genome editing via *Agrobacterium*-delivered CRISPR/Cas9
• Acceleration of generation advancement with embryo culture

## Graphical Abstract

**Figure:**
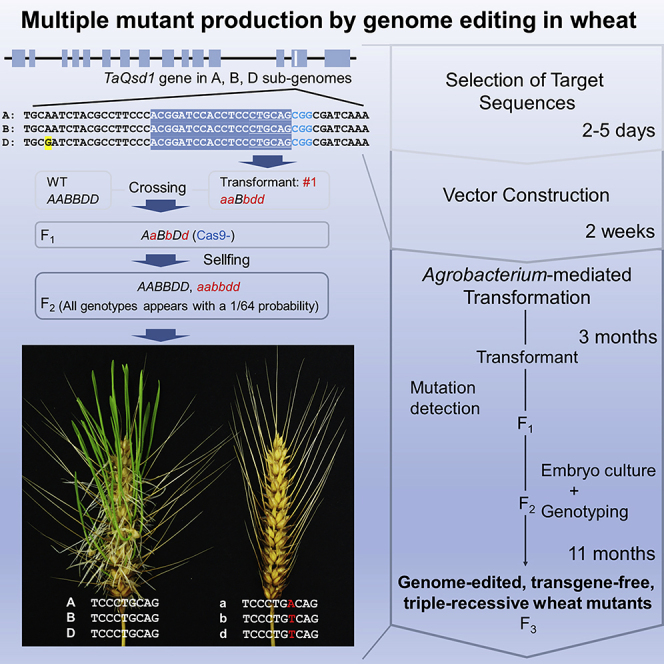

## Before You Begin

Facilities: Transformation of wheat requires immature embryos as explants; therefore, researchers must first grow wheat plants. Due to the lower efficiency of genome editing in wheat compared with in model species, we recommend using facilities that can continuously grow wheat plants in appropriate conditions throughout the year.

### Growing Wheat Plants to Obtain Immature Embryos

**Timing: about 15 weeks (depending on the season)**

**Figure 1:**
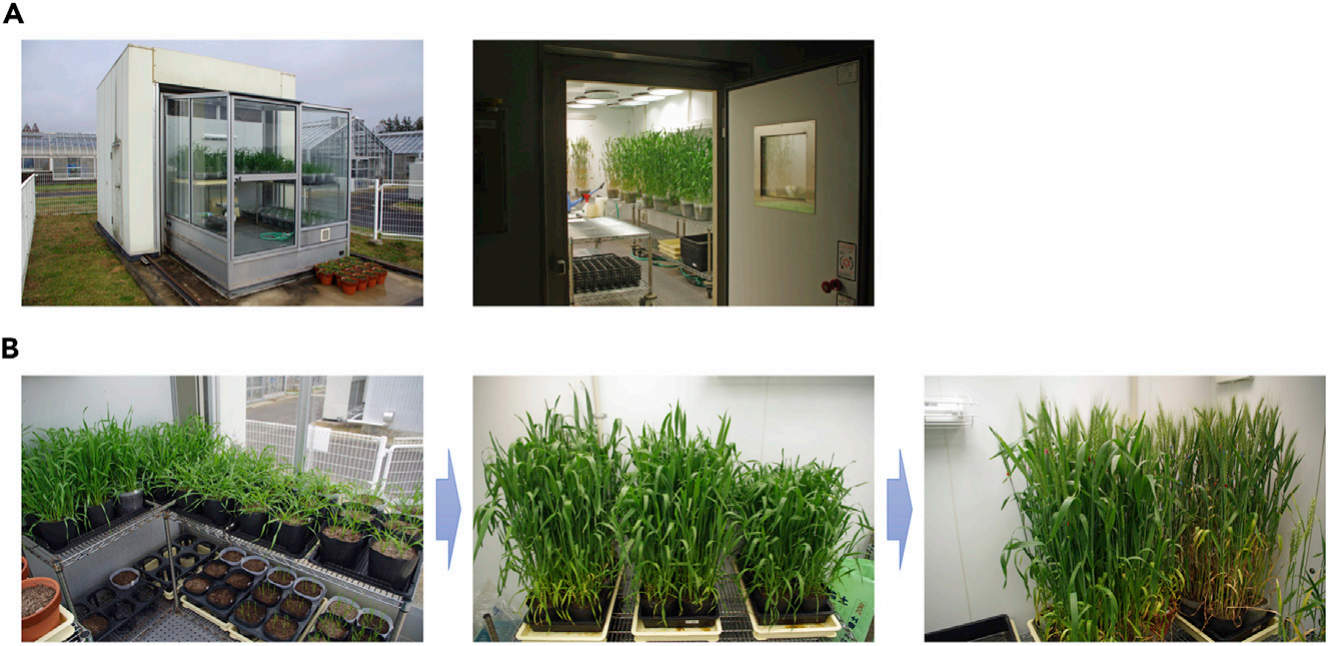
Growing Wheat Plants to Obtain Immature Embryos (A and B) (A) Facilities for growing wheat plants and (B) wheat plants growing in the facility.

1. Sow 6 seeds of wheat cv. Fielder in 18-cm plastic pots containing a 2:1 mixture of Sakata Supermix A (fine peat moss) and Nippi fertilized granulated soil. In one pot, add 2.6 L of the soil mixture and 2.6 g of the controlled release fertilizer Osmocote Exact Mini 3-4M 15-9-11+2MgO+TE.
2. Grow the plants in a glasshouse (Figure 1A left) at day/night temperatures of 16°C/10°C under an 8 h light/16 h dark photoperiod. After 10–12 weeks (depending on the season), transfer the plants just before heading to a controlled environmental chamber (Figure 1A right) with adjusted day/night temperatures of 20°C/13°C under a 14h light (300–500 μmol m^−2^ s^−1^)/10h dark photoperiod without artificial humidity control.

***Note:*** Here is an example of the conditions we use for growing wheat plants. Growing wheat plants are shown in Figure 1B. To generate a continuous supply of embryos, we recommend sowing new batches of seeds every week.

**CRITICAL:** The quality of the immature embryos is one of the most important factors for achieving efficient wheat transformation. Suitable embryos are obtained only from vigorously growing plants grown in a well-conditioned growth chamber. A disease-free plant growth environment is recommended for the effective infection of explants with *Agrobacterium tumefaciens*.

## Key Resources Table

**Table:** 

REAGENT or RESOURCE	SOURCE	IDENTIFIER
**Bacterial and Virus Strains**		

*Agrobacterium tumefaciens* EHA101	Hood et al. (1986)	N/A

**Chemicals, Peptides, and Recombinant Proteins**		

(+)-Biotin	Fujifilm-Wako	Cat# 029-08713
*myo*-Inositol	Fujifilm-Wako	Cat# 094-00281
Glycine	Fujifilm-Wako	Cat# 075-00731
Thiamine hydrochloride	Fujifilm-Wako	Cat# 201-00852
Pyridoxine hydrochloride	Fujifilm-Wako	Cat# 163-05402
Nicotinic acid	Fujifilm-Wako	Cat# 142-01232
Acetosyringone (AS)	Sigma-Aldrich	Cat# D134406
CuSO_4_·5H_2_O	Fujifilm-Wako	Cat# 039-04412
AgNO_3_	Fujifilm-Wako	Cat# 194-00832
MgCl_2_·6H_2_O	Fujifilm-Wako	Cat# 135-00165
2,4-Dichlorophenoxyacetic acid (2,4-D)	Sigma-Aldrich	Cat# D7299
Dimethyl sulfoxide (DMSO)	Fujifilm-Wako	Cat# 043-07216
Ethanol	Fujifilm-Wako	Cat# 057-00456
Picloram	Sigma-Aldrich	Cat# D5575
NaOH	Fujifilm-Wako	Cat# 194-18865
L(+)-Ascorbic acid	Fujifilm-Wako	Cat# 014-04801
Carbenicillin disodium salt	Nacarai tesque	Cat# 07129-14
Cefotaxime sodium salt	Fujifilm-Wako	Cat# 030-16113
Zeatin	Sigma-Aldrich	Cat# Z0164
HCl	Fujifilm-Wako	Cat# 080-10035
Indole-3-butyric acid (IBA)	Sigma-Aldrich	Cat# I5386
Ampicillin sodium	Fujifilm-Wako	Cat# 012-23303
Spectinomycin dihydrochloride pentahydrate	Sigma-Aldrich	Cat# S4014
Kanamycin sulfate	Fujifilm-Wako	Cat# 113-00343
CaCl_2_·2H_2_O	Fujifilm-Wako	Cat# 033-25035
Glycerol	Fujifilm-Wako	Cat# 075-00616
Tryptone	BD	Cat# 211705
Yeast extract	BD	Cat# 212750
NaCl	Fujifilm-Wako	Cat# 191-01665
Peptone	BD	Cat# 211677
D(-)-Mannitol	Fujifilm-Wako	Cat# 133-00845
L-Glutamic acid	Fujifilm-Wako	Cat# 072-00501
KH_2_PO_4_	Fujifilm-Wako	Cat# 169-04245
MgSO_4_·7H_2_O	Fujifilm-Wako	Cat# 131-00405
Murashige and Skoog plant salt mixture	Fujifilm-Wako	Cat# 392-00591
D(+)-Glucose	Fujifilm-Wako	Cat# 049-31165
2-(*N*-morpholino)-ethanesulfonic acid (MES)	Fujifilm-Wako	Cat# 345-01625
Agarose, type I	Sigma-Aldrich	Cat# A6013
L(+)-Glutamine	Fujifilm-Wako	Cat# 072-00523
Casein hydrolysate	Duchefa-Biochemie	Cat# C1301.0250
D(+)-Maltose monohydrate	Fujifilm-Wako	Cat# 130-00615
Hygromycin B	Sigma-Aldrich	Cat# 10843555001
Sucrose	Fujifilm-Wako	Cat# 196-00015
Phytagel	Sigma-Aldrich	Cat# P8169
Agar for plant culture medium	Fujifilm-Wako	Cat# 016-11875
Agar for bacterial culture medium	Fujifilm-Wako	Cat# 010-08725
Liquid nitrogen	N/A	N/A
Sodium hypochlorite	Fujifilm-Wako	Cat# 197-02206

**Critical Commercial Assays**		

High-Fidelity restriction enzyme, *Bbs*I-HF	New England Biolabs	Cat# R3539L
TAE buffer	Nacarai tesque	Cat# 32666-81
GelRed	Biotium	Cat# 41003
Restriction enzyme, *Asc*I	New England Biolabs	Cat# R0558L
Restriction enzyme, *Pac*I	New England Biolabs	Cat# R0547L
High-Fidelity restriction enzyme, *Pst*I-HF	New England Biolabs	Cat# R3140M
CutSmart Buffer	New England Biolabs	Cat# B7204S
*E. coli* DH5α Competent Cells Kit	TaKaRa Bio	Cat# 9057
QIAquick Gel Extraction Kit	Qiagen	Cat# 28706
QIAprep Spin Miniprep Kit	Qiagen	Cat# 27106
Rapid DNA Ligation Kit	Sigma-Aldrich	Cat# 11635379001
DNA polymerase, *Ex Taq* Hot Start Version	TaKaRa Bio	Cat# RR006A
Guide-it Complete sgRNA Screening System	TaKaRa Bio	Cat# 632636
Sanger sequencing kit, BigDye Terminator v3.1 Cycle Sequencing Kit	Thermo Fisher Scientific	Cat# 4337454

**Experimental Models: Organisms/Strains**		

*Triticum aestivum* cv. Fielder wild type	National Bio Resource Project_Japan	Accession No. KT020-061

**Oligonucleotides**		

Forward oligo DNA for gRNA *TaQsd1* target_1: GTTGACGGATCCACCTC CCTGCAG	Abe et al. (2019)	N/A
Reverse oligo DNA for gRNA *TaQsd1* target_1: AAACCTGCAGGGAGG TGGATCCGT	Abe et al. (2019)	N/A
Primer for sequencing of gRNA plasmid, OsU6-2F: TGCTGGAATTGCCCT TGGATCATGAACCAA	Endo et al. (2016)	N/A
Primer for detection of transgene, *Cas9* forward: TATCACCATGCCCATGAC GCTTATC	Abe et al. (2019)	N/A
Primer for detection of transgene, *Cas9* reverse: TTTCCACCTTGGCTACTAC CAACAC	Abe et al. (2019)	N/A
Primer for detection of transgene, *hpt* forward: GTGTCACGTTGCAAGACCTG	Abe et al. (2019)	N/A
Primer for detection of transgene, *hpt* reverse: GATGTTGGCGACCTCGTATT	Abe et al. (2019)	N/A
Primer for amplification of *TaQsd1* target site in three sub-genomes, TaQsd1-ABD forward: CAGCCTGGAGGGAATGACC	Abe et al. (2019)	N/A
Primer for amplification of *TaQsd1* target site in three sub-genomes, TaQsd1-ABD reverse: ACCTGGTGGAATCCAGAGC	Abe et al. (2019)	N/A
Primer for amplification of *TaQsd1* target site in A sub-genome, TaQsd1-A forward: CACATTGTCAACAAGCACACCA	Abe et al. (2019)	N/A
Primer for amplification of *TaQsd1* target site in A sub-genome, TaQsd1-A reverse: GGAGCAAAATGAGTGAATCCGTA	Abe et al. (2019)	N/A
Primer for amplification of *TaQsd1* target site in B sub-genome, TaQsd1-B forward: CTGGCCCTCATGTGGTCTTC	Abe et al. (2019)	N/A
Primer for amplification of *TaQsd1* target site in B sub-genome, TaQsd1-B reverse: GGGATCATCGCCTTGATCTTG	Abe et al. (2019)	N/A
Primer for amplification of *TaQsd1* target site in D sub-genome, TaQsd1-D forward: CATACGCACTGCCTCCTTTTCA	Abe et al. (2019)	N/A
Primer for amplification of *TaQsd1* target site in D sub-genome, TaQsd1-D reverse: GTTTCGCCCAGACACCTTTGTT	Abe et al. (2019)	N/A

**Recombinant DNA**		

pU6gRNA-oligo	Mikami et al. (2015)	N/A
pZH_gYSA_PubiMMCas9	Abe et al. (2019)	N/A

**Other**		

Soil, Sakata Supermix A	Sakata seeds	N/A
Soil, Nippi fertilized granulated soils	Nihon Hiryo	N/A
Fertilizer, Osmocote Exact Mini 3-4M 15-9-11+2MgO+TE	HYPONeX JAPAN	N/A
Sterilization filter for 1 L medium, Vacuum Filter/Storage Bottle System	Corning	Cat# 431098
Sterilization syringe filter for DMSO solution, 0.2 μm pore RC (regenerated cellulose) membrane	Corning	Cat# 431222
Sterilization filter for ~50 mL stock, Steriflip, 0.22 μm pore PES (polyethersulfone) membrane	Millipore	Cat# SCGP00525
Petri dishes (90Φ×20 mm high)	Sumitomo Bakelite	Cat# MS-13900
Petri dishes (60Φ×15 mm high)	Corning	Cat# 430589
96-well PCR plates	BM Bio	Cat# PCR-96-I
Micropore surgical tape	3M JAPAN	Cat# 1530-0
2-mL centrifuge tubes	Eppendorf	Cat# 0030120094
Scalpel blade	Futaba	No. 19
Magnetic stirrer with heater	EYELA	RCH-3
Autoclave	TOMY SEIKO	LSX-500
Thermal Cycler	Thermo Fisher Scientific	MiniAmp plus
Centrifuge	TOMY SEIKO	MDX-310
Heating water bath	TAITEC	SD-miniN
Gel electrophoresis apparatus	Takara Bio	Mupid-exU
Microvolume UV spectrophotometer	Thermo Fisher Scientific	NanoDrop One
Sanger sequencer	Applied Biosystems	3130xl DNA Analyzers
Bio-shaker	TAITEC	BR23-FP-MR
Laminar flow clean bench	Hitachi	CCV-1306E
Stereoscopic microscope	Olympus	SZ-61
Incubator (23°C, 25°C)	Panasonic	MIR-154
Growth chamber, 25°C for regeneration and rooting culture	TOMY SEIKO	CLE-305
Tissue homogenizer	Qiagen	TissueLyser II

## Resource Availability

### Lead Contact

Further information and requests for resources and reagents should be directed to and will be fulfilled by the Lead Contact, Fumitaka Abe (fabe@affrc.go.jp).

### Materials Availability

Wheat seeds used in this study are available from the National BioResource Project-Wheat, Japan (www.nbrp.jp). Plasmids used in this study are available from the Lead Contact with a completed Materials Transfer Agreement.

### Data and Code Availability

This study did not generate any unique datasets or code.

## Materials and Equipment

***Note:*** Agarose type I and Hygromycin B are the specific chemicals that we recommend should be used. Alternatives should not be substituted.

***Note:*** To excise the embryo axis from the immature embryos, a Futaba No. 19 is more suitable as a scalpel blade, but a Feather No. 14 can be used as well.

### Make Stock Solutions

**Timing: 1–2 days**

#### Biotin (0.1 mg/mL)

Dissolve 1 mg (+)-Biotin in 10 mL of distilled water, and store in 0.1-mL aliquots at −20°C in the dark.

**Table:** Modified Murashige and Skoog (MS) Vitamin Stock (1,000×)

Reagent	Weight/Volume
*myo*-Inositol	10 g
Glycine	200 mg
Thiamine hydrochloride	100 mg
Pyridoxine hydrochloride	50 mg
Nicotinic acid	50 mg
ddH_2_O	
Total	100 mL

Store in 1-mL aliquots at −20°C in the dark.

#### Acetosyringone (AS, 100 mM)

Dissolve 196.2 mg acetosyringone in 10 mL of dimethyl sulfoxide (DMSO). Sterilize with a 0.2-μm regenerated cellulose filter, and store in 1-mL aliquots at −20°C.

#### CuSO_4_ (100 mM)

Dissolve 249.7 mg CuSO_4_·5H_2_O in 10 mL of distilled water, and store in 0.1-mL aliquots at −20°C.

#### AgNO_3_ (100 mM)

Dissolve 169.9 mg AgNO_3_ in 10 mL of distilled water. Sterilize with a 0.22-μm polyethersulfone (PES) filter, and store in 0.1-mL aliquots at −20°C in the dark.

#### MgCl_2_·6H_2_O (250 mg/mL)

Dissolve 25 g MgCl_2_·6H_2_O in 100 mL of distilled water. Autoclave at 121°C for 20 min, and store at 4°C.

#### 2,4-Dichlorophenoxyacetic acid (2,4-D, 1 mg/mL)

Dissolve 50 mg 2,4-dichlorophenoxyacetic acid in 50 mL of ethanol. Sterilize with a 0.22-μm PES filter, and store at −20°C in the dark.

#### Picloram (2.2 mg/mL)

Add 1 N NaOH dropwise to 110 mg picloram until completely dissolved. Make up to 50 mL with distilled water. Sterilize with a 0.22-μm PES filter, and store in 1-mL aliquots at −20°C.

#### Ascorbic Acid (100 mg/mL)

Dissolve 5 g L(+)-ascorbic acid in 50 mL of distilled water. Sterilize with a 0.22-μm PES filter, and store in 1-mL aliquots at −20°C in the dark.

#### Carbenicillin (250 mg/mL)

Dissolve 5 g carbenicillin disodium salt in 20 mL of distilled water. Sterilize with a 0.22-μm PES filter, and store in 1-mL aliquots at −20°C in the dark.

#### Cefotaxime (250 mg/mL)

Dissolve 5 g cefotaxime sodium salt in 20 mL of distilled water. Sterilize with a 0.22-μm PES filter, and store in 1-mL aliquots at −20°C in the dark.

#### Zeatin (5 mg/mL)

Add 1 N HCl dropwise to 250 mg zeatin until completely dissolved. Make up to 50 mL with distilled water. Sterilize with a 0.22-μm PES filter, and store in 1-mL aliquots at −20°C in the dark.

#### Indole-3-butyric Acid (IBA, 1 mg/mL)

Add 1 N NaOH dropwise to 50 mg indole-3-butyric acid until completely dissolved. Make up to 50 mL with distilled water and store at 4°C.

#### Ampicillin (50 mg/mL)

Dissolve 1 g ampicillin sodium in 20 mL of distilled water. Sterilize with a 0.22-μm PES filter, and store in 1-mL aliquots at −20°C in the dark.

#### Spectinomycin (15 mg/mL)

Dissolve 300 mg spectinomycin dihydrochloride pentahydrate in 20 mL of distilled water. Sterilize with a 0.22-μm PES filter, and store in 1-mL aliquots at −20°C in the dark.

#### Kanamycin (50 mg/mL)

Dissolve 1 g kanamycin sulfate in 20 mL of distilled water. Sterilize with a 0.22-μm PES filter, and store in 1-mL aliquots at −20°C in the dark.

#### CaCl_2_ (20 mM)

Dissolve 147 mg CaCl_2_·2H_2_O in 50 mL of distilled water. Sterilize with a 0.22-μm PES filter, and store at 4°C.

#### 60% (v/v) Glycerol

Measure out 60 mL of glycerol. Make up to 100 mL with distilled water. Autoclave at 121°C for 20 min, and store at 20–25°C.

### Make Media

**Timing: 1–2 days**

**Table:** LB Medium

Reagent	Weight/Volume
Tryptone	10 g
Yeast extract	5 g
NaCl	10 g
ddH_2_O	
Total	1 L

Adjust pH to 7.0. Make up the volume to 1 L, sterilize with the Vacuum Filter/Storage Bottle System, and store at 4°C.

**Table:** YEP Medium

Reagent	Weight/Volume
Peptone	10 g
Yeast extract	10 g
NaCl	5 g
ddH_2_O	
Total	1 L

Adjust pH to 7.0. Make up the volume to 1 L, sterilize with the Vacuum Filter/Storage Bottle System, and store at 4°C.

**Table:** MG/L Medium

Reagent	Weight/Volume
Mannitol	5 g
L-Glutamic acid	1 g
KH_2_PO_4_	250 mg
NaCl	100 mg
MgSO_4_·7H_2_O	100 mg
Tryptone	5 g
Yeast extract	2.5 g
Biotin (0.1 mg/mL)	10 μL
ddH_2_O	
Total	1 L

Adjust pH to 7.0. Make up the volume to 1 L, sterilize with the Vacuum Filter/Storage Bottle System, and store at 4°C.

**Table:** Embryo Collection (Wheat MS-Based Medium-Liquid; WMS-liq) Medium

Reagent	Weight/Volume
MS basic salts	0.46 g
Modified MS vitamin stock (1,000×)	0.1 mL
Glucose	10 g
2-(*N*-morpholino)-ethanesulfonic acid (MES)	500 mg
ddH_2_O	
Total	1 L

Adjust pH to 5.8. Make up the volume to 1 L, sterilize with the Vacuum Filter/Storage Bottle System, and store at 4°C.

#### Inoculum (WMS-inf) Medium

Immediately before use, add 1 μL of 100 mM AS to 1 mL of WMS-liq medium.

**Table:** Co-cultivation (WMS-AS) Medium

Reagent	Weight/Volume
MS basic salts	0.46 g
Modified MS vitamin stock (1,000×)	0.1 mL
Glucose	10 g
MES	500 mg
CuSO_4_ (100 mM)	50 μL
ddH_2_O	
Adjust pH to 5.8	
Agarose (type I)	8 g
Make up the volume to 1 L, and autoclave at 121°C for 20 min	
Cool to 50°C, and then add the reagents below	
AS (100 mM)	2 mL
AgNO_3_ (100 mM)	50 μL

Pour 20 mL of medium into a 90-mm petri dish, and air-dry in the laminar flow clean bench for 1 h. Seal with parafilm and store at 4°C in the dark. The medium can be stored for up to 6 weeks.

**Table:** Resting (WMS-Res) Medium

Reagent	Weight/Volume
MS basic salts	4.6 g
Modified MS vitamin stock (1,000×)	1 mL
L-Glutamine	500 mg
Casein hydrolysate	100 mg
MgCl_2_·6H_2_O (250 mg/mL)	3 mL
Maltose	40 g
MES	1.95 g
ddH_2_O	
Adjust pH to 5.8	
Agarose (type I)	5 g
Make up the volume to 1 L, and autoclave at 121°C for 20 min	
Cool to 50°C, and then add the reagents below	
2,4-D (1 mg/mL)	0.5 mL
Picloram (2.2 mg/mL)	1 mL
Ascorbic acid (100 mg/mL)	1 mL
Carbenicillin (250 mg/mL)	1 mL
Cefotaxime (250 mg/mL)	0.4 mL
AgNO_3_ (100 mM)	50 μL

Pour 20 mL of medium into a 90-mm petri dish, and air-dry in the laminar flow clean bench for 1 h. Seal with parafilm and store at 20–25°C in the dark. The medium can be stored for up to 3 weeks.

**Table:** First Selection (WMS-H15) Medium

Reagent	Weight/Volume
MS basic salts	4.6 g
Modified MS vitamin stock (1,000×)	1 mL
L-Glutamine	500 mg
Casein hydrolysate	100 mg
MgCl_2_·6H_2_O (250 mg/mL)	3 mL
Maltose	40 g
MES	1.95 g
ddH_2_O	
Adjust pH to 5.8	
Agarose (type I)	5 g
Make up the volume to 1 L, and autoclave at 121°C for 20 min	
Cool to 50°C, and then add the reagents below	
2,4-D (1 mg/mL)	0.5 mL
Picloram (2.2 mg/mL)	1 mL
Ascorbic acid (100 mg/mL)	1 mL
Carbenicillin (250 mg/mL)	1 mL
AgNO_3_ (100 mM)	50 μL
Hygromycin (50 mg/mL)	0.3 mL

Pour 25 mL of medium into a 90-mm petri dish, and air-dry in the laminar flow clean bench for 1 h. Seal with parafilm and store at 20–25°C in the dark. The medium can be stored for up to 3 weeks.

**Table:** Second Selection (WMS-H30) Medium

Reagent	Weight/Volume
MS basic salts	4.6 g
Modified MS vitamin stock (1,000×)	1 mL
L-Glutamine	500 mg
Casein hydrolysate	100 mg
MgCl_2_·6H_2_O (250 mg/mL)	3 mL
Maltose	40 g
MES	1.95 g
ddH_2_O	
Adjust pH to 5.8	
Agarose (type I)	5 g
Make up the volume to 1 L, and autoclave at 121°C for 20 min	
Cool to 50°C, and then add the reagents below	
2,4-D (1 mg/mL)	0.5 mL
Picloram (2.2 mg/mL)	1 mL
Ascorbic acid (100 mg/mL)	1 mL
Carbenicillin (250 mg/mL)	1 mL
AgNO_3_ (100 mM)	50 μL
Hygromycin (50 mg/mL)	0.6 mL

Pour 25 mL of medium into a 90-mm petri dish, and air-dry in the laminar flow clean bench for 1 h. Seal with parafilm and store at 20–25°C in the dark. The medium can be stored for up to 3 weeks.

**Table:** Regeneration (WMSZ-H30) Medium

Reagent	Weight/Volume
MS basic salts	4.6 g
Modified MS vitamin stock (1,000×)	1 mL
Sucrose	20 g
MES	500 mg
CuSO_4_ (100 mM)	100 μL
ddH_2_O	
Adjust pH to 5.8	
Phytagel	3 g
Make up the volume to 1 L, and autoclave at 121°C for 20 min	
Cool to 50°C, and then add the reagents below	
Zeatin (5 mg/mL)	1 mL
Carbenicillin (250 mg/mL)	0.5 mL
Hygromycin (50 mg/mL)	0.6 mL

Pour 20 mL of medium into a 90-mm petri dish, and air-dry in the laminar flow clean bench for 1 h. Seal with parafilm and store at 20–25°C in the dark. The medium can be stored for up to 3 weeks.

**Table:** Rooting (WMSF-H15) Medium

Reagent	Weight/Volume
MS basic salts	4.6 g
Modified MS vitamin stock (1,000×)	1 mL
Sucrose	15 g
MES	500 mg
IBA (1 mg/mL)	100 μL
ddH_2_O	
Adjust pH to 5.8	
Phytagel	3 g
Make up the volume to 1 L, and autoclave at 121°C for 20 min	
Cool to 50°C, and then add the reagent below	
Hygromycin (50 mg/mL)	0.3 mL

Pour 20 mL of medium into a 90-mm petri dish, and air-dry in the laminar flow clean bench for 1 h. Seal with parafilm and store at 20–25°C in the dark. The medium can be stored for up to 3 weeks.

**Table:** Half-Strength MS Medium

Reagent	Weight/Volume
MS basic salts	2.3 g
Modified MS vitamin stock (1,000×)	0.5 mL
Sucrose	15 g
ddH_2_O	
Adjust pH to 5.8	
Agar for Plant Culture Medium	8 g
Total	1 L

Autoclave at 121°C for 20 min. Pour 25 mL of medium into a 90-mm petri dish, and air-dry in the laminar flow clean bench for 1 h. Seal with parafilm and store at 20–25°C in the dark. The medium can be stored for up to 2 months.

## Step-By-Step Method Details

### Selection of Target Sequences in Wheat

**Timing: 2–5 days**

To produce genome-edited multiple-recessive mutants in wheat, the target sequences must have a protospacer adjacent motif (PAM) for Cas9 nuclease and a restriction site for verifying the mutations. Moreover, the target sites must be present in all homoeologs, i.e. A, B, and D genomes of hexaploid wheat, for efficient cleavage and the absence of off-target sites. To select target sequences:

1. Align the three homoeologous sequences for the gene of interest. The gene information for wheat cv. Chinese Spring is available at the International Wheat Genome Sequencing Consortium (https://www.wheatgenome.org/Tools-and-Resources) website.
2. Identify 20-nucleotide target sequences within the gene of interest. When SpCas9 is employed, the target site of gRNA must be the 20-bp preceding a PAM (5′-NGG-3′) recognized by Cas9. The websites for gRNA design are useful, for example CRISPR-direct (http://crispr.dbcls.jp/) and WheatCrispr (https://crispr.bioinfo.nrc.ca/WheatCrispr/).
3. Check the predicted secondary structures of the single-stranded RNA, including the target site and scaffold of the Cas9 nuclease, at the RNAstructure website (https://rna.urmc.rochester.edu/RNAstructureWeb/), the RNAfold web server (http://rna.tbi.univie.ac.at/cgi-bin/RNAWebSuite/RNAfold.cgi), or the Quickfold web server (http://unafold.rna.albany.edu/?q=DINAMelt/Quickfold).

***Note:*** According to Liang et al. (2016), three stem-loop structures, i.e. loops RAR, 2, and 3, are crucial for gRNA stability and activity. Therefore, a gRNA that can create such a structure should be chosen. We also confirmed the importance of the predicted secondary structure of designed gRNA in wheat (Kamiya et al., 2020).

4. Check for potential off-target sites at CRISPR-P 2.0 (http://crispr.hzau.edu.cn/cgi-bin/CRISPR2/CRISPR). Reject gRNAs with potential off-target sites.
5. Conduct *in vitro* cleavage assays to exclude inferior sgRNAs, using the Guide-it Complete sgRNA Screening System. All steps should be performed according to the manufacturer’s instructions.
6. Design primer sets for amplifying the fragments, including the target sites. Websites for primer design are useful, for example Primer3Web (https://primer3plus.com/primer3web/primer3web_input.htm).

***Note:*** The success of genome editing depends on the proper function of the designed gRNA. Although the nucleotide sequences that are conserved among homoeologs in cv. Chinese Spring are almost identical to those in cv. Fielder, the target sites of cv. Fielder should be sequenced to confirm this. The whole-genome shotgun data for the null-segregant, cv. Fielder (negative control), and the transgene-positive T_1_ plant #1-8 (positive control) have been deposited in the DDBJ Sequence Read Archive under BioProject Accession PRJDB7455 (Abe et al. 2019). The sequence of Fielder can be generated by mapping whole-genome shotgun data of cv. Fielder (negative control) onto the reference target sequence (e.g. cv. Chinese Spring) to be edited. The candidate target site is better to have a restriction enzyme site that overlaps with the fourth nucleotide from the PAM; this makes it easier to detect mutations by PCR-RFLP using the restriction enzyme. If this site is not available, the PCR products, including the target sites of all homoeologs, should be sequenced to detect the mutations.

### Vector Construction

**Timing: 2 weeks**

Details of the construction method differ, depending on the vectors used to express Cas9 and the gRNA. We describe an example of the vector construction strategies used in Abe et al. 2019.

#### Cloning a gRNA into the Vector pU6gRNA-oligo

7. Synthesize forward and reverse oligo DNAs. These oligo DNAs contain the 20-bp designed sequence and must include overhangs specific for the *Bbs*I-digested gRNA cloning vector. For example, for pU6gRNA-oligo and *TaQsd1* target_1 (5′-ACGGATCCACCTCCCTGCAG-3′, Abe et al. 2019), oligo DNAs are designed as below.
5′-gttgACGGATCCACCTCCCTGCAG-3′
3′-TGCCTAGGTGGAGGGACGTCcaaa-5′
8. Digest 1 μg of the gRNA cloning plasmid, pU6gRNA-oligo, using 1 μL of *Bbs*I restriction enzyme and 2 μL of CutSmart buffer in a 20 μL final volume, and incubate at 37°C for 3 h.

**Pause Point:** The digestion may be incubated for 16–24 h.

9. Run the digested products on a 1% agarose gel in TAE buffer, and purify the digested vector using a QIAquick Gel Extraction Kit.
10. Measure the concentration of the digested vector using a microvolume UV spectrophotometer, and dilute the digested vector to 10 ng/μL.
11. Combine 1 μL of each oligo DNA (100 μM) and 48 μL of sterilized distilled water. Boil for 5 min and leave at 20–25°C for 20 min to anneal the oligonucleotide.
12. Ligate the annealed gRNA oligonucleotide into the digested vector using a Rapid DNA Ligation Kit.

**Table:** 

Reagent	Volume (μL)
Digested vector (10 ng/μL)	4
Annealed oligonucleotide	4
5 × DNA dilution buffer	2
2 × T4 DNA ligation buffer	10
T4 DNA ligase	1
Total	21

Incubate at 20–25°C for 30 min.

13. Transform 5 μL of the ligation reaction using an *E. coli* DH5α Competent Cells Kit. Plate the cells onto LB agar containing 50 mg/L ampicillin, and incubate at 37°C for 16 h.
14. Inoculate four colonies into separate 3-mL aliquots of LB medium containing 50 mg/L ampicillin and grow the cells at 37°C with vigorous shaking for 16 h.
15. Extract plasmid DNA with a QIAprep Miniprep kit.
16. Sequence plasmids with the OsU6-2F (5′-TGCTGGAATTGCCCTTGGATCATGAACCAA-3′) primer using a BigDye Terminator v3.1 Cycle Sequencing Kit to verify that the clones harbor the designed gRNA.

#### Cloning a gRNA Expression Cassette into the Binary Vector

17. Digest 2 μg of the binary vector, pZH_gYSA_PubiMMCas9, and the completed gRNA vector using 1 μL each of the restriction enzymes *Asc*I and *Pac*I, and 2 μL of CutSmart buffer in a 20 μL final volume, and incubate at 37°C for 3 h.

**Pause Point:** The digestion may be incubated for 16–24 h.

18. Run the digested products on a 1% agarose gel in TAE buffer, and purify the digested vector and gRNA expression cassette using a QIAquick Gel Extraction Kit.
19. Quantify the digested vector and gRNA expression cassette concentration using a microvolume UV spectrophotometer.
20. Ligate the digested gRNA insert into the digested binary vector using a Rapid DNA Ligation Kit.

**Table:** 

Reagent	Volume (μL)
Digested vector (30 ng/μL)	4
gRNA cassette (2.5 ng/μL)	4
5 × DNA dilution buffer	2
2 × T4 DNA ligation buffer	10
T4 DNA ligase	1
Total	21

Incubate at 20–25°C for 30 min.

21. Transform 5 μL of the ligation reaction using an *E. coli* DH5α Competent Cells Kit. Plate the cells on LB agar containing 75 mg/L spectinomycin, and incubate at 37°C for 16 h.
22. Inoculate four colonies into separate 5-mL aliquots of LB medium containing 75 mg/L spectinomycin and grow the cells at 37°C with vigorous shaking for 16 h.
23. Extract plasmid DNA with a QIAprep Miniprep kit.
24. Sequence plasmids with the OsU6-2F primer using a BigDye Terminator v3.1 Cycle Sequencing Kit to verify that the clones harbor the designed gRNA.

#### Transform the Binary Vector into *A. tumefaciens*

25. Inoculate *A. tumefaciens* strain EHA101 (Hood et al., 1986) into 10 mL of YEP medium containing 50 mg/L kanamycin and grow the cells at 28°C with vigorous shaking for 30 h.
26. Collect bacterial cells by centrifugation and resuspend in 1 mL of pre-cooled 20 mM CaCl_2_.
27. Freeze aliquots of 100 μL in liquid nitrogen. The rest can be stored at −80°C.
28. Add 0.5–1.0 μg of plasmid DNA to the frozen cells directly, and thaw at 37°C for 5 min.
29. Dilute in 1 mL of YEP medium, and shake at 28°C for 2 h.
30. Plate the cells onto YEP agar containing 75 mg/L spectinomycin, and incubate at 28°C for 2 days.
31. Pick a single spectinomycin-resistant *A. tumefaciens* colony and culture it in 1 mL of YEP medium containing 75 mg/L spectinomycin at 28°C with vigorous shaking for 20–24 h.
32. Add 1 mL of 60% (v/v) glycerol to the culture, and vortex thoroughly.
33. Store aliquots of 20 μL at −80°C.

### *Agrobacterium*-Mediated Transformation

**Timing: 3 months**

*Agrobacterium*-mediated transformation is a straightforward approach to plant transformation that results in the insertion of only one or a few copies of the transgene. Producing the transgene-free genome-edited mutant is efficiently accomplished by crossing the resulting transformed plant with a wild-type plant and screening for progeny that contain the mutation but lack the transgene in the F_2_ generation.

#### Infection of Immature Embryos with *A. tumefaciens*

34. Add 10 μL of *A. tumefaciens* glycerol stock (step 33) to 10 mL of MG/L medium (no antibiotics), and grow cells at 28°C with vigorous shaking for 20–24 h.
35. Collect bacterial cells by centrifugation and resuspend to a cell density of 0.4 of A_660_ in WMS-inf medium.
36. Collect immature seeds at the right developmental stage from panicles about 15 days after anthesis (DAA) (Video S1).

**CRITICAL:** The use of immature embryos at the correct developmental stage is a critical factor, and the size of the embryos is a very good indicator of the stage. Immature embryos that are 2.0–2.5 mm in length along the long axis are optimal for transformation. The time (DAA) required for embryos to reach the optimum stage differs, depending on the genotype and the season. Because wounding of immature embryos is detrimental for infection by *A. tumefaciens* and subsequent tissue culture, handle the immature embryos carefully up to the step for excising the embryo axis (step 50).

37. Remove the glumes, lemma, and palea with forceps (Video S1).
38. Sterilize immature seeds with 70% ethanol for 1 min and 0.5% sodium hypochlorite for 15 min, and then wash three times with sterilized distilled water.
39. Isolate immature embryos from the immature seeds under a stereoscopic microscope and transfer the embryos into 2.0 mL of WMS-liq medium in a 2.0-mL microcentrifuge tube (Video S1). Taking into account subsequent handling of the embryos, it is recommended to place fewer than 100 embryos into one microcentrifuge tube.
40. Invert the microcentrifuge tube several times and remove the medium.
41. Add 1.9 mL of WMS-liq medium.
42. Centrifuge the microcentrifuge tube with a fixed-angle rotor with a maximum radius of 83 mm at 20,000×*g* at 4°C for 10 min.
43. Remove the medium from the microcentrifuge tube and add 1.0 mL inoculum (step 35) (Video S1).
44. Invert the microcentrifuge tube frequently for 30 s (Video S1).
45. Incubate at 20–25°C for 5 min.
46. Transfer the immature embryos to WMS-AS medium using a 60-mm petri dish. Although the inoculum will be absorbed by the culture medium, any excess inoculum should be removed with forceps (Video S1).

***Note:*** About 50 embryos can be placed onto a single plate. We recommend starting with 10 embryos per plate and increasing the number up to 50 embryos depending on the level of skill. When the plate is left open for a long time to excise the embryo axis (step 50), the embryo will gradually dry out and die.

47. Gently turn over the immature embryos with the scutellum-side upwards.
48. Seal the plate with Parafilm.
49. Incubate the plate at 23°C in the dark for 2 days.

Video S1. Infection of Immature Embryos with *A. tumefaciens* (Refer to Steps 36, 37, 39, 43, 44, and 46 in *Agrobacterium*-Mediated Transformation)

#### Tissue Culture after Co-cultivation

50. Excise the embryo axis from the immature embryos using a scalpel and forceps (Video S2).

51. Transfer the immature embryos to WMS-Res medium with the scutellum side up. About 100 embryos can be placed onto a single plate. Seal the plate with micropore surgical tape and culture at 25°C in the dark for 5 days.
52. Transfer immature embryos to WMS-H15 medium. About 25 embryos can be placed onto a single plate. Culture at 25°C in the dark for 2 weeks.
53. Transfer proliferated explants in WMS-H30 medium. Tissues derived from up to 25 embryos can be placed onto a single plate. Culture under the same conditions for 3 weeks.
54. Transfer proliferated explants to WMSZ-H30 medium. Tissues derived from up to 25 embryos can be placed onto a single plate. Seal the plate with Parafilm and culture at 25°C under a 14h light (90 μmol m^−2^ s^−1^)/10h dark photoperiod for 2 weeks.
55. Transfer all regenerated green shoots to WMSF-H15 medium (Video S3) and culture under the same conditions for 2 weeks.

Video S2. Excising the Embryo Axis from the Immature Embryos after Co-cultivation with *A. tumefaciens* (Refer to Step 50 in *Agrobacterium*-Mediated Transformation)

56. Change Parafilm to micropore surgical tape and culture for 3–5 more days.

***Note:*** During embryo culture (steps 51 to 53), the plates are sealed with micropore surgical tape. During the regeneration and rooting (steps 54 and 55), the plates are sealed with Parafilm. During the final step of rooting, we change the Parafilm to micropore surgical tape, and then culture for the final 3 to 5 days. The change from Parafilm to micropore surgical tape 3–5 days before transplanting helps the rooted plants acclimate to the new growth environment.

57. Transplant rooted hygromycin-resistant plants to soil (Video S4).

Video S3. Transferring Regenerated Shoots to the Rooting Medium (Refer to Step 55 in *Agrobacterium*-Mediated Transformation)

***Note:*** Most individuals that regenerate and root under this selection condition are transformants.

58. Extract DNA from the leaves of plants.
59. Confirm the presence of the transgene by PCR with *hpt* and *Cas9* primer sets (Key Resources Table).

Video S4. Transplanting Rooted Plants to Soil (Refer to Step 57 in *Agrobacterium*-Mediated Transformation)

### Detection and Genotyping of the Mutation

**Timing: 3 days**

60. Amplify the targeted region of the transformants by PCR with the conserved primer set that recognizes all three homoeologs (TaQsd1-ABD in Key Resources Table).
61. Digest 5 μL of the PCR products using 0.5 μL of *Pst*I enzyme with 1 μL of CutSmart buffer in a 10-μL final volume. Incubate at 37°C for 3 h.
62. Run the products on a 2% agarose gel with Gel Red.
63. Visualize the results on a UV transilluminator.
64. Identify which homoeologs contain a mutation using three specific primer sets (such as TaQsd1-A, TaQsd1-B, and TaQsd1-D in Key Resources Table), one for each homoeolog.
65. Sequence the undigested fragments of three specific primer sets using a BigDye Terminator v3.1 Cycle Sequencing Kit to identify the type of mutation.

### Acceleration of Generation Advancement with Embryo Culture

**Timing: 2 weeks**

Isolating immature embryos and germinating them on medium, rather than waiting for seeds to mature, allows the next generation of plants to be obtained more rapidly. This technique can also be applied to immature seeds resulting from crosses.

66. Aseptically isolate immature embryos from wheat plants 13–15 DAA and place them on half-strength MS medium with the embryo axis side upwards. Seal the plates with Parafilm.
67. Incubate at 25°C under a 14 h light/10 h dark photoperiod for 7–10 days.
68. Change Parafilm to micropore surgical tape 3–5 days before transplanting, to help the rooted plants acclimate to the new growth environment.
69. Transplant rooted plants to soil.
70. Extract DNA from the leaves of plants.
71. Confirm the segregation of the transgene by PCR with *hpt* and *Cas9* primer sets, and identify which homoeologs contain a mutation using three specific primer sets as described above.

***Note:*** Using this technique, we successfully produced a transgene-free triple-recessive spring wheat mutant in a short time. Only 14 months were required from the infection of immature embryos to obtaining the homozygous F_3_ mutant seeds of a cross progeny (Abe et al., 2019).

## Expected Outcomes

Genome-edited, transgene-free, triple-recessive mutants can be produced in hexaploid wheat. We confirmed that the transgene, which was located in a maximum of five loci, can be segregated away simultaneously by crossing with the wild-type plant in the next (F_1_) generation (Abe et al., 2019). In our conditions, the transformation efficiency of treated immature embryos ranged between 2–5%, and 7–39% of the transformants, depending on the target sequence, all contained mutations at any of the target sites (Kamiya et al., 2020).

## Limitations

An efficient *Agrobacterium*-mediated transformation technique (Ishida et al., 2015) is currently available only for cv. Fielder and a limited range of alternative cultivars. Protocols are limited to investigating the roles of genes in those genotypes. Mutations could be transferred from these cultivars to another by standard crossing. Our technique for generation advancement via embryo culture can also be used to accelerate the introduction of multiple mutations into other genetic backgrounds.

The genome sequence of wheat (IWGSC (The International Wheat Genome Sequencing Consortium), 2018) helps to precisely identify T-DNA fragments within the genome. We mapped four T-DNA integrations to their respective chromosomal positions of sub-genomes by sequencing the border junctions of the T-DNA and the wheat genome. This approach was quite difficult before the release of the wheat reference genome, due to the sequence similarity among homoeologs.

The high-quality genome sequence of the target haplotype might be needed as an initial step for genome editing in wheat. We previously sequenced bacterial artificial chromosome clones from cv. Chinese Spring harboring wheat orthologs of barley (*Hordeum vulgare*) *Qsd1* to re-sequence one in cv. Fielder and to design experiments for genome editing.

We also examined whether this *Agrobacterium*-based genome editing system is generally applicable for other genes. We reproduced our results in wheat cv. Fielder with other genes. However, we had limited success for mutation induction, and obtained mutations only in a few genes with a lower frequency of multiple mutations in a single plant. For example, we attempted to mutate targeted wheat orthologs of barley *SD2* (*TaQsd2*), but were not successful (unpublished data).

## Troubleshooting

### Problem

The transformation success rate is low.

### Potential Solution

The wheat embryos are very sensitive to environmental conditions. If the transformation success rate is low, check the plant growth conditions before investigating other aspects of the protocols, such as vectors and media compositions. We recommend testing the steps for infection of explants with *A. tumefaciens* using a reporter gene such as β*-GLUCURONIDASE* (*GUS*). Monitoring the level of expression of a transgene in immature embryos at the resting culture stage, 7 days after infection with *A. tumefaciens*, provides useful information for optimizing the transformation protocol.

### Problem

Genome-edited plants cannot be obtained.

### Potential Solution

The success of genome editing depends on the proper functioning of the designed gRNA. If genome-edited plants cannot be obtained, please redesign the gRNA first. Deep learning-based systems to predict CRISPR/Cas9 sgRNA on-target cleavage efficiency, such as DeepSpCas9 (http://deepcrispr.info/DeepSpCas9/) and DeepHF (http://www.deephf.com/index/#/Predict), may help you to select target sequences.

If a different vector construct is used, please check the promoters for gRNA. We confirmed that the two promoters, *OsU6* (Feng et al., 2013) and *TaU6* (Shan et al., 2014), can appropriately drive gRNA expression in wheat.

## References

[bib1] Abe F., Haque E., Hisano H., Tanaka T., Kamiya Y., Mikami M., Kawaura K., Endo M., Onishi K., Hayashi T., Sato K. (2019). Genome-edited triple-recessive mutation alters seed dormancy in wheat. Cell Rep..

[bib2] Endo M., Nishizawa-Yokoi A., Toki S. (2016). Targeted mutagenesis in rice using TALENs and the CRISPR/Cas9 system. Methods Mol. Biol..

[bib3] Feng Z., Zhang B., Ding W., Liu X., Yang D., Wei P., Cao F., Zhu S., Zhang F., Mao Y., Zhu J. (2013). Efficient genome editing in plants using a CRISPR/Cas system. Cell Res..

[bib4] Hood E.E., Helmer G.L., Fraley R.T., Chilton M.D. (1986). The hypervirulence of Agrobacterium tumefaciens A281 is encoded in a region of pTiBo542 outside of T-DNA. J. Bacteriol..

[bib5] Ishida Y., Tsunashima M., Hiei Y., Komari T. (2015). Wheat (Triticum aestivum L.) transformation using immature embryos. Methods Mol. Biol..

[bib6] IWGSC (The International Wheat Genome Sequencing Consortium) (2018). Shifting the limits in wheat research and breeding using a fully annotated reference genome. Science.

[bib7] Kamiya Y., Abe F., Mikami M., Endo M., Kawaura K. (2020). A rapid method for detection of mutations induced by CRISPR/Cas9-based genome editing in common wheat. Plant Biotechnol..

[bib8] Liang G., Zhang H., Lou D., Yu D. (2016). Selection of highly efficient sgRNAs for CRISPR/Cas9-based plant genome editing. Sci. Rep..

[bib9] Mikami M., Toki S., Endo M. (2015). Comparison of CRISPR/Cas9 expression constructs for efficient targeted mutagenesis in rice. Plant Mol. Biol..

[bib10] Shan Q., Wang Y., Li J., Gao C. (2014). Genome editing in rice and wheat using the CRISPR/Cas system. Nat. Protoc..

